# Dynamics of Vagal Activity Due to Surgery and Subsequent Rehabilitation

**DOI:** 10.3389/fnins.2019.01116

**Published:** 2019-11-05

**Authors:** Vincent Grote, Zoran Levnajić, Henry Puff, Tanja Ohland, Nandu Goswami, Matthias Frühwirth, Maximilian Moser

**Affiliations:** ^1^Human Research Institute, Weiz, Austria; ^2^Orthopedic Rehabilitation Center, Humanomed Center Althofen, Althofen, Austria; ^3^Division of Physiology, Otto Loewi Research Center for Vascular Biology, Immunology and Inflammation, Medical University of Graz, Graz, Austria; ^4^Complex Systems and Data Science Lab, Faculty of Information Studies in Novo Mesto, Novo Mesto, Slovenia

**Keywords:** circadian rythm, surgery, vagal tone, inflammatory control, rehabilitation

## Abstract

**Background:**

Vagal activity is critical for maintaining key body functions, including the stability of inflammatory control. Its weakening, such as in the aftermatch of a surgery, leaves the body vulnerable to diverse inflammatory conditions, including sepsis.

**Methods:**

Vagal activity can be measured by the cardiorespiratory interaction known as respiratory sinus arrhythmia or high-frequency heart-rate variability (HRV). We examined the vagal dynamics before, during and after an orthopedic surgery. 39 patients had their HRV measured around the period of operation and during subsequent rehabilitation. Measurements were done during 24 h circadian cycles on ten specific days. For each patient, the circadian vagal activity was calculated from HRV data.

**Results:**

Our results confirm the deteriorating effect of surgery on vagal activity. Patients with stronger pre-operative vagal activity suffer greater vagal withdrawal during the peri-operative phase, but benefit from stronger improvements during post-operative period, especially during the night. Rehabilitation seems not only to efficiently restore the vagal activity to pre-operative level, but in some cases to actually improve it.

**Discussion:**

Our findings indicate that orthopedic rehabilitation has the potential to strengthen the vagal activity and hence boost inflammatory control. We conclude that providing a patient with a vagal reinforcement procedure *prior* to the surgery (“pre-habilitation”) might be a beneficial strategy against post-operative complications. The study also shows the clinical usefulness of quantifying the cardiorespiratory interactions.

## Introduction

Vagus nerve is the longest nerve of the autonomic nervous system (ANS) and the key component of parasympathetic nervous system. Its baseline activity, often refered to as *vagal activity*, is crucial for maintaining several body functions at rest, including heart, lungs, and digestion. Vagal activity is stronger during sleep, stabilizing the body’s circadian rhythms, which are key for good general health (Mundigler et al., 2002; Moser et al., 2006a; Kastner et al., 2010; Rocha et al., 2011; Scheiermann et al., 2013; Curtis et al., 2014; Papaioannou et al., 2014; Alamili, 2015; Madrid-Navarro et al., 2015; Wright et al., 2015).

Vagal activity is also the critical factor behind the functionality of the *inflammatory reflex*, mechanism responsible for resolving the inflammation once its purpose has been served (Tracey, 2002, 2007; Moser et al., 2008; Rosas-Ballina and Tracey, 2009; Leslie, 2014; Mayer, 2015). Actually, the vagal inflammatory reflex involves 80% vagal afferents and 20% efferents, which means that four times more information is collected by the brain than transmitted to the periphery: macrophages in the inflamed tissue produce inflammation signals such as TNF-alpha and interleukin 1 (Andersson and Tracey, 2012; Olofsson et al., 2012), which attract other monocytes from nearby blood vessels. Vagal afferents carry receptors for these signals and communicate with certain stem brain areas transmitting the information on inflammation location and strength (Andersson and Tracey, 2012). Upon processing this information, vagal efferents respond by release of acetylcholine at the location of the inflamed tissue (Olofsson et al., 2012). Nicotinergic acetylcholine receptors have been identified on the surface of the macrophages, which down-regulate their cytosine production as a response to the cholinergic stimulation (Rosas-Ballina and Tracey, 2009), thereby reducing the attraction of additional inflammatory immune cells. This inflammatory reflex loop prevents over-activity of the immune system enabling the brain to locally control the immune activity. It also represents the “first line” of inflammation control (Olofsson et al., 2012).

In addition to improving the effectiveness and usefulness of the inflammatory reflex, a strong vagal activity protects against several serious or chronic conditions. They include atherosclerosis, ulceral colitis, Hashimoto’s disease, type 2 diabetes, cancer (Donchin et al., 1992; Moser et al., 2006a; Das, 2011; Huston and Tracey, 2011; Chow et al., 2014), and sepsis, which is know to be related to weakening of body’s natural ability to resolve the inflammation (Tracey, 2002; Nguyen et al., 2006; Nathan and Ding, 2010). The vagus nerve can be electrically and pharmacologically stimulated, while its overall activity can be improved via acupuncture, nutritional therapies, and physical exercise (Huang et al., 2005; Moser et al., 2017).

Measuring vagal activity can be reliably done by analyzing the heart rate variability (HRV). In fact, HRV is created by the interaction between ANS and the sinus node of the heart (Moser et al., 1995). Its main component, originating in vagal activity, is respiratory modulation of the heart frequency. Through a gating process that takes place in the brainstem, vagal activity is responsible for speeding up the heart when we breath in, and slowing it down when we breath out (Langhorst et al., 1983). The amplitude of this respiratory sinus arrhythmia is proportional to the vagal activity. This cardiorespiratory interaction mediated by the vagus nerve is faster (approximately 0.25 Hz) than other influences of the ANS (0.1 Hz or slower). Actually, postsynaptic vagal activity is mediated by acetylcholine, which is rapidly degraded in the synaptic gap by its esterase, an enzyme that warrants fast decay of neurotransmitters after release. This makes the parasympathetic synapses much faster than the sympathetic ones, which use norepinephrine postsynaptically (Moser et al., 1998). Norepinephrine is eliminated mainly by presynaptic reuptake, which results in transmitters remaining longer in the synaptic gap. In short, the intensity of this cardiorespiratory interaction can be used as a reliable measure of vagal activity (Moser et al., 1994).

Surgery is a situation where it is paramount to preserve the strong vagal activity. There is aboundant evidence that surgical procedures weaken the vagal activity (Donchin et al., 1992; Munford and Tracey, 2002; Williamson et al., 2010), while surgery is a notorious trigger of sepsis. With this in mind we performed a clinical study aimed at testing the effects of surgery on patient’s vagal activity. Our study relies on a longitudinal measurement and comparison of circadian dynamics (24 h recordings) of vagal activity in patients before, during and after a surgical procedure. This includes measurements during and after rehabilitation, for up to 1 year after the surgery. Vagal activity is computed from HRV data as described in Moser et al. (1994), Moser et al. (1995), Lehofer et al. (1999).^1^ We report our results in what follows.

## Materials and Methods

### Patients and Ethics

Thirty-nine patients (23 female of age 32–83 and 16 male of age 32–81) were recruited for our study. They were hospitalized at the Orthopedic Rehabilitation Center at Humanomed Center in Althofen, Austria for total endoprosthetic orthopedic surgery (replacement of hip or knee joints). Inclusion criteria were age 30–90 and completion of 3 week rehabilitation within 3 months after the surgery. Exclusion criteria were usage of pacemaker and clinically identified complications (thrombosis, pulmonary embolism, or wound healing disorders). Patients were informed about the nature and the purpose of the study, signed the informed consent and participated voluntarily. After the study, personal results were given to all patients with adequate expert explanation. The study was authorized by the Ethical Committee of the Carinthian Government, authorization number A 02/05, 01 February, 2005. Methods were chosen in accordance with the relevant guidelines and regulations.

### Measurement Protocol

In order to investigate the behavior of vagal activity in relation to the surgery and the subsequent recovery, we divided the operation-rehabilitation process into the following four phases: immediately before the surgery (*pre-operative phase*), immediately after the surgery (*peri-operative phase*), *rehabilitation*, and long-term recovery (*post-operative phases*). On ten specific days each patient had his/her vagal activity measured over the entire day, i.e., the 24 h circadian cycle. These are referred to as “measurement days” and denoted as T1,T2,…T10. They are “time periods” chosen to best reflect each phase of the operation-rehabilitation process as follows.

• *Immediately before the surgery* (*“pre-operative*”; measurement days T1 and T2). Patients were measured on 2 days, sometime between 8 and 2 days prior to the surgery.
• *Immediately after the surgery* (*“peri-operative*”; measurement day T3). Patients were measured on 1 day sometime between 2nd and 4th day after the surgery, depending on their availability due to their medical state.
• *Rehabilitation* (*“post-operative*”; measurement days T4, T5, and T6). Patients were measured on 2nd, 9th, and 16th day of the inpatient rehabilitation process (rehabilitation started 26.6 ± 11.3 days after the surgery).
• *Long-term recovery* (measurement days T7, T8, T9, and T10). Patients were measured at the beginning of 6th, 12th, 26th, and 52nd week after the end of the rehabilitation.

**FIGURE 1:**
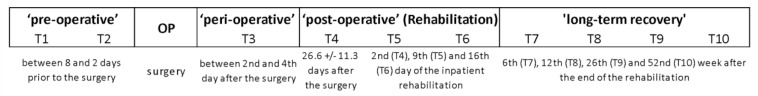
Shematic representation of the division of operation-rehabilitation process into phases and measurement days for the purposes of our study.

For better orientation we show in Figure 1 the schematic representation of this division.

Since previous studies found a strong cure-treatment effects to peak after 6 weeks (Moser et al., 1998; Lehofer et al., 1999), we used this time period to perform the first post-rehabilitation measurements. After this, we used approximate doubles of 6-week-intervals until the end after 1 year, which is a reasonable (almost) exponential frame for observing long-term effects. These measurements are taken on as equidistant time-points as patients compliance allowed.

### HRV Measurements

On each measurement day we made precise circadian measurements of heart-rate variability (HRV) for each patient. That is to say, each patient had his/her instantaneous heart rate recorded continuously for 24 h, using a mobile 8000 Hz Holter-ECG with 16 bit A/D converter (ChronoCord, manufacturer: Joysys, Austria), developed from space medical research (Gallasch et al., 1997). The instrument was attached to a patient in a way not to interfere with his/her regular daily activities. Vagal activity was computed from these time series of around 100,000 heartbeats per patient/day as described below, and then averaged over 5 min intervals distributed evenly over 24 h. After this averaging, one circadian time series consisted of 1440 values, i.e., 1 value per each minute of the measurement day. Thus, on each measurement day we obtained for each patient a circadian time series with 1440 HRV values. We defined the *circadian time* from noon on a measurement day to noon on the following day. Our study lasted for over an entire year (408 ± 34,3 days) for each patient (not all patients participated simultaneously). 16 of them completed all the measurements (age 32–81, 11 female). For these 16 patients, some data points were still missing (13.75%). We report the data here only from these 16 patients.

### Pre-processing and Computation of the Vagal Activity

Pre-processing steps included filtering and removal of the artifacts, done according to Lehofer et al. (1999). R peaks were detected from the ECG recordings by a digital filter described in Moser et al. (1994), Lehofer et al. (1999) to more than 1 ms accuracy. We then computed the vagal activity time series from the cardiorespiratory arrhythmia by the robust time-domain method named *logRSArr*. The method is described and evaluated in Moser et al. (1994), Lehofer et al. (1999) and its relaton with cardiorespiratory interactions is established in Topçu et al. (2018). In short, we used the formula:

$$\mathrm{Vagal activity}=\log_{10}(m\,e\,d\,i\,a\,n_{\left(5\,\min\right)}\,\left|R\,R_{i+1}-R\,R_{i}\right|),$$

where RRs are the consecutive inter-beat (RR) intervals, and the median value is taken over the 5 min interval. This logRSArr method acts as a filter emphasizing high-frequency HRV components, and reflects the vagally mediated respiratory component of HRV better than RMSSD or high frequency HRV (Topçu et al., 2018). Also, the chosen method is more robust than frequency-domain methods and allows a higher time-resolution. Robustness is here important since it prevents the results from disturbances by movement artifacts and ectopic heartbeats. Upon computation, we focused our analysis on these data, which consist of one circadian time series of vagal activity values for each patient on each measurement day.

### Statistical Analysis

General linear models (GLM) were used to perform a per protocol analysis via repeated measures ANOVA. Within-subject factor is “time period” [pre-operative (individual means of T1 and T2), peri-operative (T3), rehabilitation (T4, T5, and T6), and long-term recovery (T7, T8, T9, and T10)] for three different “activity periods” of vagal activity within a day (logRSArr during “sleep,” “wake,” and “24 h mean”). The calculation of the used periods “sleep” and “wake” is based on visual controlled activity protocols of the patients, whereby transitions between wake and sleep, the first and last 30 min of each activity period, were not taken into account. For these statistical analyses, missings (in already aggregated values) in “time period” had to be replaced by individual means of nearby time points in 11 out of 192 cases (5.7%). Later we add pre-operative “vagal-type” as between-subject factor, computed via median split of aggregated 24 h means of logRSArr from T1 and T2 (pre-operative vagal activity) to quantify a hypothesized interaction (time course x vagal-type) for a different development in time course of subjects with a constitutional high vs. low vagal activity.

## Results

### Overall Circadian Dynamics of Vagal Activity

**FIGURE 2:**
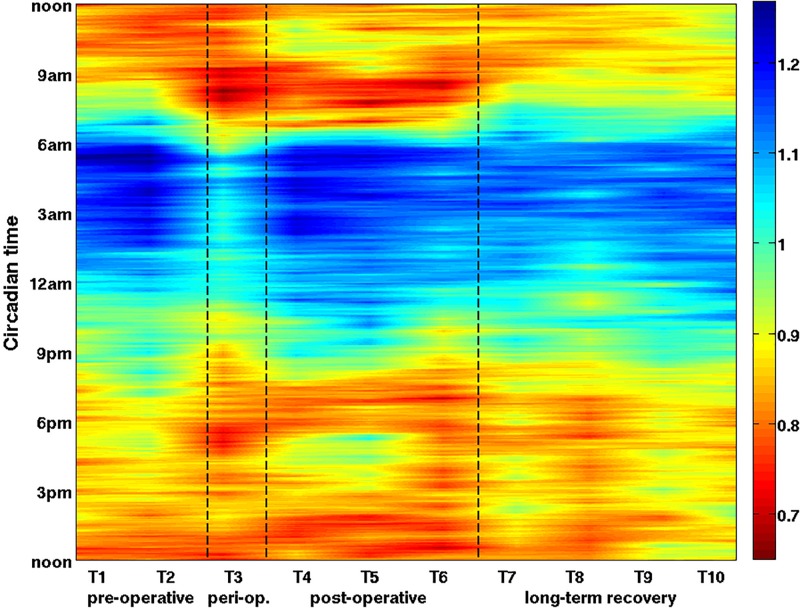
The overall dynamics of the vagal activity during the operation-rehabilitation process. Each color represents the value of the vagal activity averaged over all 16 patients on a given measurement day (horizontal axis, T1–T10) and at a given circadian hour (vertical axis, time runs from bottom to top). Clinical phases are also indicated on the horizontal axis. Each averaged vagal activity value is represented as a color, where red means low and blue means high vagal activity (see color bar). Phases of the operation-rehabilitation process are delimited by the dashed lines. Vagal activity is reduced during peri-operative phase (T3), especially during the night. During the post-operative rehabilitation (T4–T6) original vagal activity values are gradually restored. See also our statistical analysis (Tables 1, 2), which confirms the statistical significance of the vagal activity changes.

We first present the overall circadian behavior of vagal activity during operation-rehabilitation process. To this end, we averaged the data over all 16 patients, obtaining one averaged circadian time series for each measurement day. The results are shown in Figure 2.

Natural oscillations of vagal activity from stronger (night) to weaker (day) are visible on all measurement days, indicated by a change of blue during the night to red during the day. Clearly, peri-operative vagal activity (T3) is severely weakened over the entire circadian cycle. In fact, on T3 a major decrease was observed even during the night, when the immune system is actually more active. During rehabilitation (T4–T6), vagal activity is gradually restored to its pre-operative circadian rhythm and to its usual circadian values. On T5 we observe a longer night time interval of strong vagal activity, which increases their average daily vagal activity. On T4–T6 we see a slight decrease between 7–9 am and 6–8 pm, most likely attributable to rehabilitation treatments. During long-term recovery (T7–T10), previously observed circadian pattern shifts to later in a day. This reduces the overall daily vagal activity, restoring the normal circadian oscillations and amplitudes, similar to pre-operative ones.

To confirm the statistical significance of these results, we performed standard ANOVA on these vagal changes and show the results in Table 1.

**TABLE 1:** Statistical significance of vagal activity differences over time.

**Repeated measures ANOVA [time (4)]^†^ logRSArr [ms]**			**Mean**	***SD***	***Post hoc* (LSD)**	***F***	***P significance***	**$\eta_{\text{p}}^{2}$**
Time (vagal activity; log RSArr)	Sleep	Pre-operative	1,152	0,175	Pre- vs. Peri-operative (*p* = 0.004^∗∗^)	4,124	0.025^∗^	0,216
		Peri-operative	1,021	0,172				
		Rehabilitation	1,106	0,233				
		Long-term recovery	1,119	0,271				
		Overall	1,099	0,195	Square time effect	12,739	0.003^∗∗^	0,459
	Wake	Pre-operative	0,887	0,182		2,189	0.102	0,127
		Peri-operative	0,808	0,183	Peri- vs. Long-term recovery (*p* = 020^∗^)			
		Rehabilitation	0,841	0,244				
		Long-term recovery	0,882	0,203				
		Overall	0,854	0,185	Square time effect	7,475	0.015^∗∗^	0,333
	Mean-24 h	Pre-operative	0,980	0,155	Pre- vs. Peri-operative (*p* = 0.028^∗^)	3,243	0.046^∗^	0,178
		Peri-operative	0,885	0,170				
		Rehabilitation	0,935	0,225	Peri- vs. Long-term recovery (*p* = 010^∗^)			
		Long-term recovery	0,967	0,212				
		Overall	0,942	0,175	Square time effect	17,095	0.001^∗∗^	0,533

*^†^*N* = 16 (13.75% replaced missing values). Marked levels of significance: ^∗^*p* < 0.05 and ^∗∗^*p* < 0.01.*

We find that the effect of the surgery on vagal activity is most significant during sleep (repeated ANOVA: part. Eta^2^ [η^2^] = 0.216; *Post hoc*_lsd_: pre- vs. peri-operative *p* = 0.004). The vagal activity recovery after surgery is most pronounced in the 24 h mean values in the long-term recovery phase after finishing the rehabilitation (0.885 ± 0.170 peri-operative vs. 0.967 ± 0.212 long-term recovery: η^2^ = 0.178; *Post hoc*_lsd_: *p* = 0.010). The dynamics can also be observed in vagal activity while the patient is awake (η^2^ = 0.127), but this is probably more confounded by various daily activities.

### Scatter Plot Analysis

We next studied more closely how peri-operative, rehabilitation (“post-operative”) and long-term recovery values of vagal dynamics depend on the corresponding pre-operative values. We investigated two specific time intervals: during the day from noon to 8 pm (when vagal activity is typically low) and during the night from 10 pm to 6 am (when vagal activity is usually strong). We averaged the values of vagal activity over these two intervals, but this time for each patient and on each measurement day separately. This provides an average daily and an average nightly vagal activity value for each patient and for each measurement day.

**FIGURE 3:**
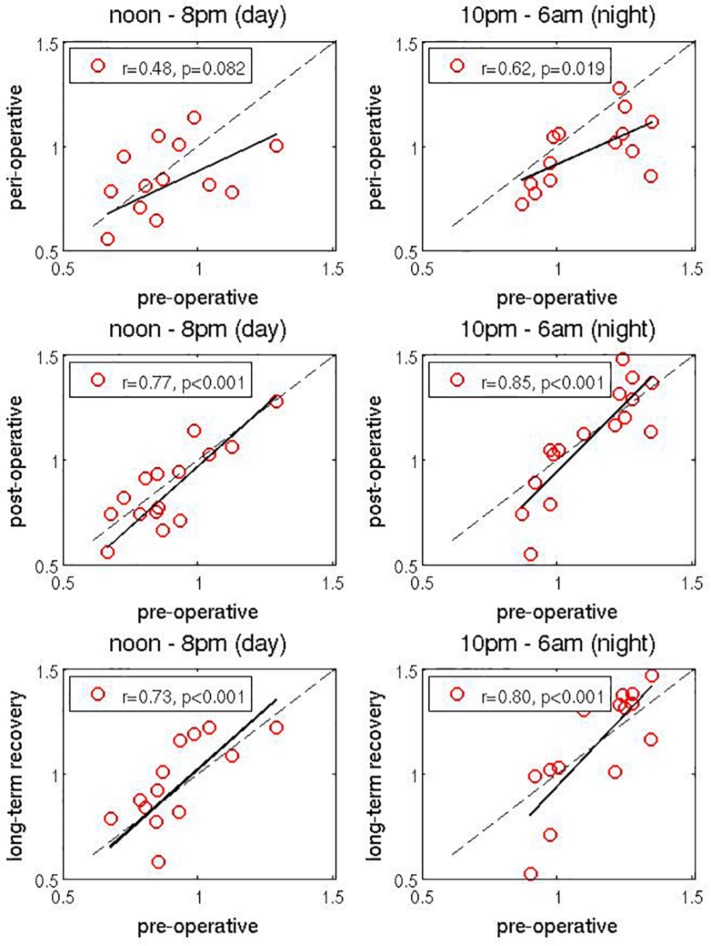
Scatter plots of vagal activity values. Peri-operative values **(top panels)**, post-operative values **(middle panels)**, and long-term recovery values **(bottom panels)** are reported on the vertical axis, as a function of pre-operative values, which are in all panels reported on horizontal axis (each dot represents one patient). They are computed, respectively, as values on T3, average between T4 and T5, average between T9 and T10, average between T1 and T2. Changes in day-time values (measured from noon to 8 pm) are shown on the left and changes in the night-time values (measured from 10 pm to 6 am) are shown on the right. Linear regression is shown as a full black line and the line of identity is shown as a dashed black line. Correlation coefficient *r* is reported for each plot separately. Significances are all *p* < 0.08. While the event of surgery clearly reduces the vagal activity, the rehabilitation process gradually restores it to the original values. In fact, in some cases final vagal activity values are actually higher than the original pre-operative values. This effect is especially pronounced during the night (due to logarithmic representation of vagal activity values, real changes are actually more pronounced than it appears). See text and Table 2. Figures have different numbers of points, since for 16 patients that completed the study certain daily and/or nightly values are missing.

First, to examine the change of vagal activity due to surgery, the peri-operative vagal activity (measured on T3) was compared to pre-operative vagal activity (taken as the mean between measurements on T1 and T2). This comparison is shown as two scatter plots in top panels on Figure 3 In both cases a reduction of vagal activity can be observed due to the surgery, especially in patients with larger pre-operative values. This is even more pronounced for the nightly values. Second, in the two middle panels in Figure 3 we repeat the same analysis, but this time for post-operative values. They were taken as the mean between measurements on T5 and T6 (we exclude T4 from this averaging to allow more time for rehabilitation to make noticable effect), and scatter ploted agains pre-operative values (as above). Both plots show that during rehabilitation, the vagal activity values are gradually restored to the pre-operative ones. This effect is very clear during the night: patients with weaker pre-operative vagal activity show a very slow recovery, whereas patients with strong pre-operative vagal activity in fact show a noticable increase of vagal activity as a result of the early rehabilitation process. Note that due to the logarithmic representation of vagal activity values (see section “Materials and Methods”), nightly increase in vagal activity is actually much higher than immediately visible in these plots, and also higher than the decrease for the patients with low pre-operative vagal activity. Third, in the bottom panels of Figure 3 we scatter plot the long-term recovery values against pre-operative values. The former were taken as the mean between T9 and T10 (again, we exclude T7 and T8 from averaging to give more time to long-term recovery). We find a generally positive slope of the regression line, indicating overall improvement of the vagal activity (recall that the logarithmic representation of vagal activity is less faithful toward larger values). Again, patients with stronger pre-operative values benefit from stronger improvement, while patients with lower pre-operative values show similar or slightly weaker values. However, we must take into account here that several months have passed since the surgery, so other life factors might have influenced the vagal activity.

**FIGURE 4:**
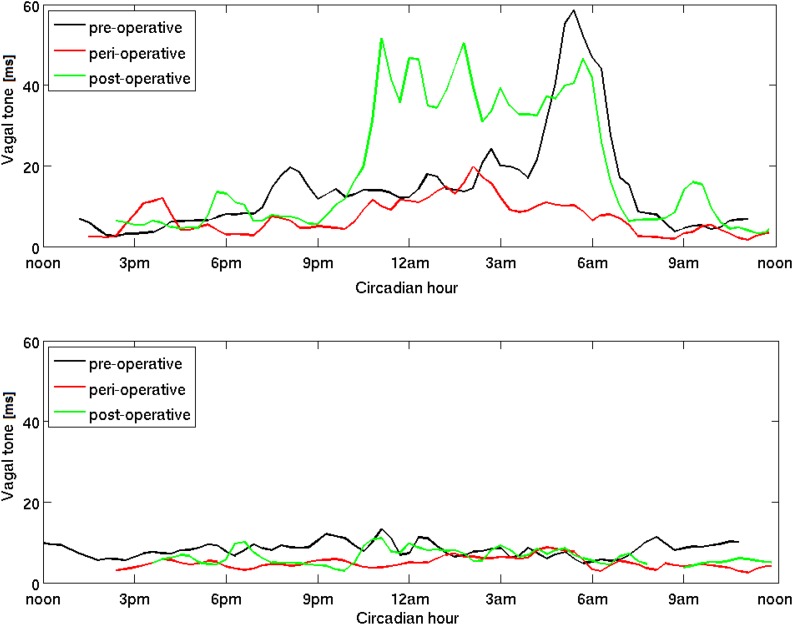
Circadian dynamics of vagal activity (vagal tone) during the operation-rehabilitation process. A patient with a strong pre-operative vagal activity is shown in the **top panel** and another patient with a weak pre-operative vagal activity is shown in the **bottom panel**. Three circadian time series for the entire 24 h cycle are shown in each panel, measured on three specific days, pre-operative on T1, peri-operative on T3 and post-operative on T5. We remove the logarithmic scale for better clarity (see section “Materials and Methods”) and use the moving average over a window of several minutes to avoid minute-to-minute fluctuation. While the patient in the **top panel** shows a clear improvement of post-operative vagal activity, especially during the night, the patient in the **bottom panel** shows very little improvement, and even during the night.

Table 2 shows the results of two-way ANOVA [within-factor (time-course; 4-stage), between-factor (pre-operative-vagal activity; 2-stage)] performed to detect differences (interactions) in time course among the patients. Interestingly, while the patients suffer from larger reduction of vagal activity due to surgery (top panels of Figure 3), patients with strong pre-operative vagal activity exhibit larger increase of vagal activity during rehabilitation (middle panels Figure 3 and Table 2: repeated MANOVA: Interaction: *p* = 0.021, η^2^ = 0.898), and actually finish with vagal activity values even higher than the pre-operative ones. This is the case, at least, in some patients. The multivariate significant increase of the vagal activity (*p* = 0.042, η^2^ = 0.869, see Table 2) after surgery is dependent on the pre-operative values (time × vagal-type: *p* = 0.021, η^2^ = 0.898). This is shown for individual cases later in Figure 4. Due to the small and heterogeneous sample of patients, this significant dependence on initial values (pre-operative) was not seen in inference statistics via univariate testing (*p* > 0.292) for the used aggregated time points.

**TABLE 2:** Statistical analysis for interaction (time × vagal-type) according (Figures 2, 3).

**Repeated measures ANOVA (*N* = 16) [4 × 2 design (time × vagal-type)]^†^ logRSArr [ms]**		**Vagal-type**				**Effects time vagal-type**	***F***	***P significance***	**$\eta_{\text{p}}^{2}$**
		
		**Strong vagal**		**Weak vagal**					
		**activity (*n* = 8)**		**activity (*n* = 8)**					
							
		**mean**	**SD**	**mean**	**SD**				
Sleep	Pre-operative	1281	0,088	1022	0,139				
	Peri-operativ	1,114	0,151	0,927	0,145	Time	4,203	0.022^∗^	0,231
	Rehabilitation	1246	0,110	0,966	0,248	Vagal-type	13,803	0.002^∗∗^	0,496
	Long-term recovery	1287	0,120	0,951	0,279				
	Overall	1,232	0,083	0,967	0,184	Interaction	1,287	0.292	0,084
Wake	Pre-operative	1,007	0,177	0,767	0,079				
	Peri-operativ	0,907	0,111	0,709	0,192	Time	2,224	0.099^(*)^	0,137
	Rehabilitation	1,007	0,155	0,675	0,201	Vagal-type	14,528	0.002^∗∗^	0,509
	Long-term recovery	1,007	0,112	0,757	0,200				
	Overall	0,982	0,118	0,727	0,148	Interaction	1,234	0.306	0,082
Mean-24 h	Pre-operative^†^	**1,097**	**0,125**	**0,863**	**0,066**				
	Peri-operativ	0,986	0,109	0,783	0,162	Time	3,273	0.048^∗^	0,190
	Rehabilitation	1,095	0,118	0,775	0,190	Vagal-type	17,151	0.001^∗∗^	0,551
	Long-term recovery	1,089	0,111	0,844	0,222				
	Overall	1,067	0,090	0,816	0,146	Interaction	1,141	0.336	0,075
	Time					Time	4,432	0.042^∗^	0,869
MANOVA	Vagal-typ					Vagal-type	5,208	0.016^∗^	0,566
	Interaction (Time × vagal-type)					Interaction	5,900	0.021^∗^	0,898

**FIGURE 5:**
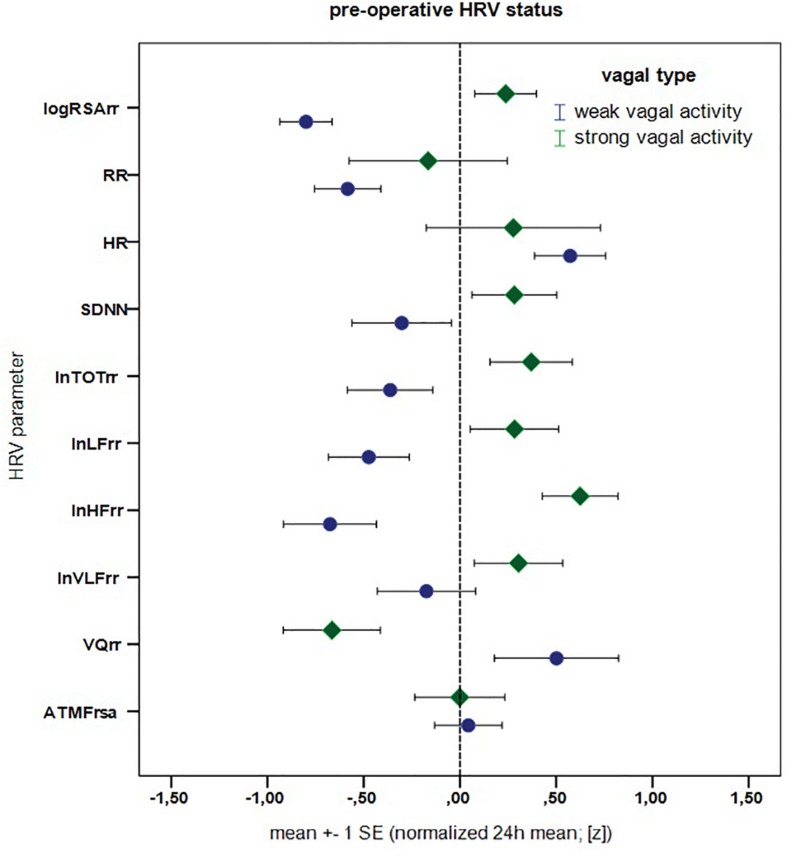
Pre-operative cardio-autonomic status - patients with weak vs. stronge vagal activity. Mean (absolute) *z*-difference (weak vs. strong vagal activity) = 0.759, *T* = –2.936 (df = 14), *p* = 0.011^∗^ (MANOVA: *F* = 3.113, *p* = 0.062, $\eta_{\text{p}}^{2}$ = 0.675, calculated for HR, SDNN, TOT, LF, HF, VLF, and VQ).

*^†^A median split of pre-operative vagal activity was used to classify into vagal-types with strong of weak vagal activity (cut-off value: 0.9654). This classification was also used for Figure 5. ^∗^*p* < 0.05 and ^∗∗^*p* < 0.01.*

### Effects on the Entire Circadian Dynamics

Finally, we investigated the effects of surgery and rehabilitation on the entire circadian dynamics of vagal activity. To that end we selected two patients, one with a generally strong and the other with generally weak pre-operative vagal activity (see also Table 2). In Figure 4 top panel, we show three circadian time series for the first patients. Both the reduction due to surgery (red) and the improvement due to rehabilitation (green) are clearly visible over almost the entire circadian cycle. During rehabilitation, the improvement is especially pronounced during the first part of the night: vagal activity increases beyond its pre-operative values. Next we examine the same time series for the second patient (with generally weak vagal activity) in Figure 4 bottom panel. Similar patterns are found over the circadian cycle, but the improvement due to rehabilitation is now almost entirely absent. Weak pre-operative vagal activity seem to be connected to weak vagal response during rehabilitation. This again confirms that the rehabilitation process can enhance vagal activity to values higher than normal, and particularly so for the patients with initially strong values. Our results suggest that if the patient’s vagal activity could be boosted pre-operatively, this patient could realistically expect a lesser risk of peri-operative sepsis and a better outcome of rehabilitation. For completeness, we later make a clearer separation between strong and weak vagal activity.

### Analysis of Other HRV Parameters

As an addition to logRSArr we next examine further HRV parameters in Table 3 over 24 h during the examined time interval. Next to a lower vagal activity (logRSArr, *p* = 0.046) and an increased heart rate (*p* = 0.072; RR: *p* = 0.011), HRV is generally reduced immediately after surgery and it takes time to recover (only in the follow-up after rehabilitation; “long-term recovery”; all *p* < 0.10). Autonomic Balance (Ratio LF/HF) is not affected by the orthopedic surgery (*p* = 0.830).

**TABLE 3:** Analysis of other HRV parameters.

**Heart rate variability (HRV)**	**Overall: *n* = 16**		**Time course**				**Unifactoriell GLM –**			***Post hoc***
***parameters***	**(24 h mean)**		**(time points; 24 h mean)**				**time effect**			**test (LSD)**
					
			**Pre-**	**Peri-**	**Rehabilitation**	**Long-term**				
	**Mean ± *SD***	**Unit**	**operative (1)**	**operative (2)**	**(3)**	**recovery (4)**	***F***	***P***	**$\eta_{\text{p}}^{2}$**	***p* < 05**
vagal activity (logRSArr)	0,94 ± 0,17	log(ms)	0,98	0,88	0,93	0,97	3,24	0.046^∗^	0,18	1 vs. 2; 2 vs. 4
Consecutive inter-beat intervals (RR)	788,67 ± 75,33	ms	790,49	760,15	794,48	809,56	4,16	0.011^∗^	0,22	2 vs. 3, 4
Heart rate (HR)	78,69 ± 7,81	bpm	78,91	80,60	78,34	76,90	2,49	0.072^(*)^	0,14	2 vs. 4
Standard deviation of RR (SDNN)	45,65 ± 9,37	ms	49,49	40,22	46,26	46,61	5,00	0.016^∗^	0,25	1 vs. 2
Total variability pow er (InTOTrr)	7,11 ± 0,45	ln(ms^2^)	7,30	6,90	7,10	7,14	3,95	0.035^∗^	0,21	1 vs. 2, 3
Low frequency power (InLFrr)	5,57 ± 0,51	ln(ms^2^)	5,76	5,31	5,58	5,63	4,65	0.019^∗^	0,24	1 vs. 2; 2 vs. 4
High frequency pow er (InHFrr)	4,37 ± 0,91	ms^2^	4,53	4,12	4,37	4,47	2,70	0.077^(*)^	0,15	1 vs. 2
Very low frequency power (InVLFrr)	6,46 ± 0,40	ms^2^	6,64	6,29	6,43	6,45	3,18	0.065^(*)^	0,17	1 vs. 2, 3
Ratio LF/HF	1,20 ± 0,60	[ ]	1,23	1,18	1,21	1,16	0,19	0.830	0,01	
Respiratory rate (ATMFrsa)	17,52 ± 1,29	fpm	17,02	17,64	17,46	17,95	3,19	0.062^(*)^	0,18	1 vs. 3,4; 3 vs. 4

Marked levels of significance: ^(*)^*p* < 0.1 and ^∗^*p* < 0.05.

Next we compare our patients with age and gender matched reference values from healthy controls at the pre-operative time. Results are reported in Table 4. Overall, patients in our clinical sample seem to have higher pre-operative heart rate (*z* = 0.43, *p* = 0.096)^2^ with a slightly reduced vagal activity (*z* = –0.28, *p* = 0.115), where the other HRV values are in general similar to healthy individuals (all *p* ≥ 0.60, MANOVA with HR, SDNN, TOT, LF, HF, VLF, VQ; pre-operative patients vs. healthy controls: *F* = 0.674, *p* = 0.675; see Table 4 and Figure 5).^3^

**TABLE 4:** Age and gender matched reference values for pre-operative HRV parameters.

**Heart rate variability (HRV)**	**Healthy matched sample (*n* = 32)^†^**		**Rehab sample (*n* = 16): “pre-operative”**		
**parameters**	**24 h mean**		**[normalized 24 h mean; (z)]**		
		
	**Mean ± *SD***	**Unit**	**mean [z] ± *SD***	***T***	***P***
vagal activity (logRSArr)	1,03 ± 0,23	log(ms)	−0,28 ± 0,67	−1,67	0.115
Consecutive inter-beat intervals (RR)	827,85 ± 99,76	ms	−0,37 ± 0,89	−1,69	0.112
Heart rate (HR)	75,08 ± 9,02	bpm	0,43 ± 0,96	1,78	0.096^(*)^
Standard deviation of RR (SDNN)	49,64 ± 14,68	ms	−0,01 ± 0,72	−0,06	0.956
Total variability power (InTOTrr)	7,30 ± 0,61	ln(ms^2^)	0,00 ± 0,70	0,02	0.982
Low frequency power (InLFrr)	5,83 ± 0,72	ln(ms^2^)	−0,10 ± 0,72	−0,53	0.604
High frequency power (InHFrr)	4,55 ± 0,92	ms^2^	−0,03 ± 0,90	−0,11	0.911
Very low frequency power (InVLFrr)	6,61 ± 0,56	ms^2^	0,06 ± 0,71	0,37	0.719
Ratio LF/HF	1,28 ± 0,55	[ ]	−0,08 ± 1,00	−0,33	0.747
Respiratory rate (ATMFrsa)	16,98 ± 2,27	fpm	0,02 ± 0,57	0,15	0.884

*^†^Age, 59.25 ± 10.34 (68.8% female).*

Using above HRV parameters, we can now make a clearer distinction between patients with strong as opposed to weak vagal activity. To this end we perform MANOVA [calculated for HR, SDNN, TOT, LF, HF, VLF, and VQ; *F* = 3.113, *p* = 0.062, *p*.Eta2 = 0.675] and report the results in Figure 5. Patients with a pre-operative “weak vagal activity” differ markedly from patients with “strong vagal activity” in almost all HRV parameters (effect size: mean absolute *z*-differences^3^ = 0.759, *p* = 0.011). This confirms that vagal activity (logRSArr) is a good indicator for differences in cardio-autonomic (HRV) status, as it reflects different pattern of HRV markers and thus types of cardio-autonomic profiles. It can also be useful as a marker for different reaction types, e.g., to surgery, possible complications like sepsis or different clinical courses. This also clarifies our choice of two patiens with weak vs. strong vagal activity in earlier Figure 4.

### Analysis of Results of Questionnaires

Further data relative to clinical information of patients (standardized questionnaires; Zerssen, 1976; Hobi, 1985; Grote, 2009) are shown in Table 5. Most patients report an improvement of subjective well-being (*p* = 0.027), already during “rehabilitation.” Only in the “long-term recovery” period, the values (“well-being” and “sleep recovery”) reach those of healthy reference data [0.00 ± 1.00; (z)^3^]. General symptoms of “complaints” appear to be less affected over time (*p* = 0.139) and remain higher than in healthy controls (*z* > 0.75) throughout the whole observation period. Hence in general, no significant correlations between autonomic (HRV) parameters and questionnaire results can be observed.

**TABLE 5:** Statistics and Questionnaires.

	**Repeated measures ANOVA questionnaires – psychometric scales [z]^†^**		**Mean^†^ ± *SD***	***Post hoc* (LSD; *p* ≤ 05)**	***F***	***P***	**$\eta_{\text{p}}^{2}$**
Time course	Well-being (*n* = 13) (Hobi, 1985)	Pre-operative (1)^‡^ Peri-operativ (2) Rehabilitation (3) Long-term recovery (4)	−0,426 ± 0,780 −0,771 ± 0,879 −0,155 ± 0,852 0,033 ± 0,686	1 vs. 4 2 vs. 3, 4	4,201	0.027^∗^	0,276
	Complaints (*n* = 14) (Zerssen, 1976)	Pre-operative (1) Peri-operativ (2) Rehabilitation (3) Long-Term recovery (4)	1,094 ± 1,147 1,094 ± 1,109 0,811 ± 1,313 0,753 ± 1,278		1,949	0.139	0,140
	Sleep recovery (*n* = 13) (Grote, 2009)	Pre-operative (1) Peri-operative (2) Rehabilitation (3) Long-Term recovery (4)	−0,253 ± 0,995 −0,802 ± 1,277 −0,258 ± 1,164 −0,018 ± 1,085	2 vs. 4	2,391	0.086^(*)^	0,179

^†^Normalized with healthy controls [*z*-values]. ^‡^Correlation with vagal activity: *r* = 0.387, *p* = 0.155. MANOVA (*n* = 12) - time: *F* = 1.846, *p*-0.071^(*)^, $\eta_{\text{p}}^{2}$ = 0.156 (Pillai). Marked levels of significance: ^(*)^*p* < 0.1 and ^∗^*p* < 0.05.

## Discussion

Using the intensity of cardio-respiratory sinus arrhythmia for determination of vagal activity, we showed that vagal activity decreases around the time of (orthopedic) surgery, and increases during rehabilitation and long-term recovery. The former is an indicator of dangers accompanying surgical procedures, including sepsis. We found that in the wake of surgery vagal activity is impaired in essentially all patients in our sample. This impairment is present during both day and night, but is more prominent during the night. The observed decrease of vagal activity implies the breakdown of the inflammatory reflex. This hinders the ability of the body to timely resolve inflammation, thus leaving the patient considerably more vulnerable to diverse inflammatory conditions after surgery. Given that surgery and the associated tissue injury are both pro-inflammatory, preserving the inflammation resistance is paramount during this critical period. Moreover, weakening of vagal activity could be unintentionally enhanced in other ways, such as via narcotic treatments that are known to dampen ANS, including its vagal component (Shapiro et al., 2010; Tarvainen et al., 2012). Our findings suggest that caution must be observed when using such narcotics.

Our next main result is that the rehabilitation process, besides being clearly effective in restoring the vagal activity, also seems to provide a way of boosting it, as suggested by the larger than normal values observed in several patients. This vagal activity increase was particularly prominent during the night, which is normally characterized by higher vagal activity compared to the day-time values. In fact, sleep is well-known to be important for general health and helpful in many medical conditions (Reynolds et al., 2012; Moser and Kripke, 2013). Therefore, rehabilitation appears to be suited for restoring the autonomic regulation and thus the inflammatory reflex, which persist even 1 year after rehabilitation in our study.

Vice versa of this situation has been reported. For instance, independent of the origin of inflammation, vagal activity is always reduced in inflammatory conditions (Lujan and DiCarlo, 2013). This may lead to a positive feedback loop or a vicious circle, entangling the inflammation reflex, and the accompanying pathology. Some forms of obesity are indeed known to lead to inflammation, while at the same time the chronic inflammation promotes obesity-associated diabetes (Wang et al., 2003). This indicates that besides in the development of sepsis, dysfunctional vagal control or circadian disturbance of the ANS could play a role in several key diseases of modern society, including cardiovascular diseases, metabolic syndrome and/or even development of cancer (Moser et al., 2006b; Eiró and Vizoso, 2012).

On the other hand, we realize that after surgery vagal activity is bound to increase, regardless of whether the patient undergoes rehabilitation or not. It is hard to identify which part of vagal activity increase that we observed comes as a result of rehabilitation, and which part can be attributed to natural bodily regeneration mechanisms. Yet there is extensive evidence for the positive influence of rehabilitation on a number of factors related to general well-being (Strauss-Blasche et al., 2004), many of which are directly associated with the strength of vagal activity. Our findings indicate that rehabilitation generally does have a positive effect on vagal activity, but the question of precise difference of vagal activity between patients that undergo rehabilitation and those that do not remains to be answered.

Is there a minimum value of vagal activity above which the patient is protected against diseases (such as sepsis)? While this interesting question calls for more research, we report that none of the patients contracted sepsis. This suggests that, at least for sepsis, this threshold value is below the minimums observed here.

### Clinical Applications of Our Findings

We suggest that in order to reduce the chances of inflammatory conditions in the wake of surgery, it might be worthwhile to provide some activities that increase the vagal activity *prior* to the surgery (“pre-habilitation”). This would increase the patient’s vagal inflammatory resistance allowing him/her to cope with the event of surgery and the associated stress more effectively (Geiss et al., 2005; Laitio et al., 2007; Mazzeo et al., 2011; Bravi et al., 2012; Bohanon et al., 2015; Ernst et al., 2017; Reimer et al., 2017; Yang et al., 2018). An additional argument in favor of this conclusion comes from our observation that patients with strong pre-surgery values make the best use of the rehabilitation in improving their vagal activity. Hence, strengthening the vagal activity of a patient during the weeks before the planned surgery appears to be a promising strategy to minimize the risk of vagal fail and hence inhibit the developement of inflammatory states. The aim of this paper was to provide more empirical evidence for these hypotheses, which if ultimately proven correct, may open new approaches, for example, in treating or preventing sepsis. This study also shows the clinical value of a quantified cardiorespiratory interactions, the respiratory sinus arrhythmia.

## Data Availability Statement

The datasets generated for this study are available on request to the corresponding author MM (max.moser@medunigraz.at).

## Ethics Statement

This study involved the patients from the Orthopedic Rehabilitation Center at Humanomed Center in Althofen, Austria. Patients were informed about the nature and the purpose of the study, signed the informed consent and participated voluntarily. After the study, personal results were given to all patients with adequate explanation from a doctor. The study was authorized by the Ethical Committee of the Carinthian Government, authorization number A 02/05, 01 February, 2005. All methods used in this study are in accordance with the relevant guidelines and regulations, usual for research in medical sciences.

## Author Contributions

MM, VG, and HP envisaged the study. HP and TO arranged for the patient voluntary participation. VG and TO carried out the measurements. MM, MF, VG, and ZL analyzed the data and the results. ZL, NG, VG, and MM wrote the manuscript. All authors reviewed the manuscript.

## Conflict of Interest

The authors declare that the research was conducted in the absence of any commercial or financial relationships that could be construed as a potential conflict of interest.

## Footnotes

1 Actually, HRV is long known as a good marker for risk stratification, early prognosis and prediction of post-operative complications.

2 Standard scores, also called *z*-values (z) are calculated by subtracting the population mean from an individual raw score and then dividing the difference by the population standard deviation (here: for healthy controls; e.g., age and gender matched reference values for autonomic (HRV) parameters see Table 4). Similarly, the deviations from zero or from mean-differences of *z*-values can interpreted as effect size as presented in Figure 5 and Table 4.

3 The data for healthy individuals were taken from a general database.
